# The effect of omega-3 fatty acid use on women's mental health in postpartum depression: a systematic review and meta-analysis study

**DOI:** 10.1590/1806-9282.20241389

**Published:** 2025-03-17

**Authors:** Sevda Karakaş, Sevda Uzun, Handan Özcan

**Affiliations:** 1 Gümüşhane University, Faculty of Health Sciences, Department of Nursing – Gümüşhane, Turkey.; 2 Health Sciences University, Faculty of Health Sciences, Department of Midwifery – İstanbul, Turkey.

## INTRODUCTION

The postpartum period is a period in which the mother experiences significant anatomical, physiological, and psychological changes as well as the transition to parenthood and assumes new roles and responsibilities^1,2^. In addition to the rapidly changing hormones and physiological state with childbirth, the responsibilities imposed on the mother, being a mother for the first time, lack of social support, lack of spousal support, and problems related to infant care can increase the risk of depression^3,4^.

Postpartum depression (PPD) causes a loss of physical and mental energy in the mother, negatively affecting her family, work, and social life and decreasing her quality of life. The worldwide prevalence of PPD is 15%, and this rate varies between 12.5 and 42.7% in Turkey^5,6^. Early diagnosis and treatment of PPD are important to protect maternal and infant health. Untreated PPD can turn into a progressive and chronic disease and cause increased mortality and morbidity in both mother and infant^7,8^. The main approach in the treatment of PPD is psychotherapy in combination with medication, but medication has many unknown effects on breast milk. The use of medication in the treatment of PPD reduces the amount of milk in the nursing mother and may cause side effects such as sedation, respiratory arrest, and neurobehavioral disorders in the infant^2^. Since the safety of drug use in the treatment of PPD is controversial, the effects of some nutrients in the prevention or alleviation of PPD are being investigated^7,8^.

It is known that adequate and balanced nutrition of the mother in the postpartum period is a basic requirement for the protection and development of mental health. It is emphasized that especially omega-3 fatty acids, vitamin B12, vitamin D, selenium, and iodine consumption are important for healthy mental health^9^. In the latest reports of the World Health Organization, the importance of seafood between pregnancy and mental health is emphasized^10^. In studies in the literature, it is recommended to take fish oils containing omega-3 fatty acids, which are an important nutrient in preventing PPD or alleviating its effects, by consuming fish or as a dietary supplement^11,12^. The aim of this study was to evaluate the effect of omega-3 fatty acid use on women's mental health in postpartum depression by systematic review and meta-analysis method.

## METHODS

This systematic review and meta-analysis study was prepared according to the PRISMA checklist [Preferred Reporting Items for Systematic Reviews and Meta-Analyses Protocols (PRISMA) Checklist]^13^. To reduce the risk of bias in this systematic review and meta-analysis, literature search, article selection, and data extraction were performed independently by three researchers. These steps were then checked again by three researchers. The quality assessment of the studies included in the systematic review and meta-analysis was performed by the researchers.

### Inclusion and exclusion criteria

In this study, data were screened according to PICOS;

- Study group (P: Patient): Women who have given birth
- Intervention (I: Intervention): Omega-3 fatty acid use
- Comparison (C: Comparison): Not using omega-3 fatty acids
- Outcomes (C: Outcomes): Depression
- Study design (S: Study design): Experimental, quasi-experimental, published in Turkish and English.

Letters to the editor and systematic and traditional reviews were excluded from the scope of this study.

### Search strategy

The search was conducted between June and July 2024 through PubMed, EBSCO host Web of Science, Yök Thesis, and Google Scholar with the keywords "omega fatty acids and postpartum depression" and "women and omega fatty acids" or "omega-3 PUFAs and postpartum depression" or "omega-3 FA or DHA or EPA or fish oil and postpartum depression", in accordance with MeSH. Since there were few studies on the use and efficacy of omega-3 fatty acids in women with postpartum depression, no year limitation was made and all years were searched.

### Selection of studies

**Figure 1:**
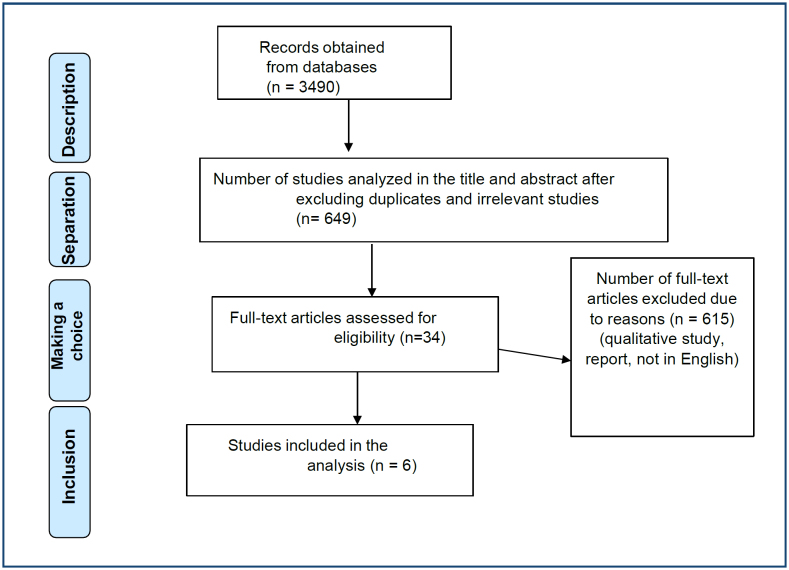
Selection of studies according to the PRISMA Preferred Reporting Items for Systematic Reviews and Meta-Analyses flow diagram.

The search initially yielded 3,490 records. After the repeated studies were removed, 649 records were examined to select the title and abstract. As a result of this review, 34 studies were selected to be examined in full text. Then, six articles whose full text was accessed were examined according to the inclusion and exclusion criteria, and six studies reporting results on the effect of omega-3 fatty acids in the postpartum period were included in the analysis^11,12,14-17^. Explanations about the selection process of the articles are given in Figure 1.

### Data extraction of study data

The data extraction tool developed by the researchers was used to obtain the research data. With the data extraction tool, data on the main findings of the studies included in the systematic review and meta-analysis such as author and publication year, study design, year, country, and sample size were collected.

### Assessment of methodological quality of studies

The quality assessment of the studies included in this systematic review and meta-analysis was conducted by The Joanna Briggs Institute with quality assessment tools prepared according to the research design. The evaluation tools used in this study were selected according to the designs of the studies included in the systematic review and meta-analysis. In our study, evaluation tools consisting of 13 questions for randomized controlled trials (The Joanna Briggs Institute Critical Appraisal Tools for Use in IBI Systematic Reviews, 2021) and 9 questions for quasi-experimental studies^18,19^ were used. The questions in these tools are answered with "Yes, No, Uncertain, Not Applicable" options.

Methodological quality was assessed independently by two authors and consensus was reached through a discussion.

In this study, the evaluation results for each study are shown in Figure 1 as "Quality score."

### Data synthesis

For the statistical calculations of this study, CMA Ver. 2. was used. The heterogeneity between the studies was evaluated with chi-squared statistics and Higgins I² tests, and an I² of more than 50% was considered to indicate significant heterogeneity. Studies with I^2^≤50% and p>0.1 were evaluated using the fixed effects model; however, studies with I^2^>50% and p>0.1 were evaluated using the random effects model.

## RESULTS

**Figure 2:**
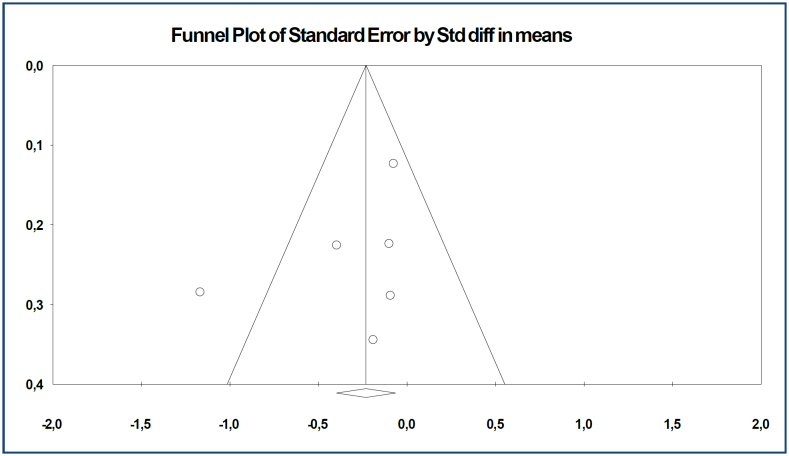
Funnel plot of studies reporting results on the effect of omega-3 fatty acid use on women's mental health in postpartum depression.

The selection of studies according to the PRISMA flow diagram in this systematic review and meta-analysis is given in Figure 2.

Six of the studies included in the study were randomized controlled experimental studies.

The total sample size of the studies was 613 (intervention group: 364; control group: 249) (Figure 1). The results are important in terms of showing that data reflecting the importance of the use of omega-3 fatty acids on mental health in the postpartum period have been revealed. In all of the studies included in this systematic review and meta-analysis, it was determined that the quality of evidence met more than 50% of the items of the assessment tool (Figure 1). This is important in terms of showing that the information presented in our systematic review and meta-analysis is based on studies with an acceptable level of evidence quality.

Meta-analysis results on the effect of omega-3 fatty acid use on women's mental health in postpartum depression

**Figure 3:**
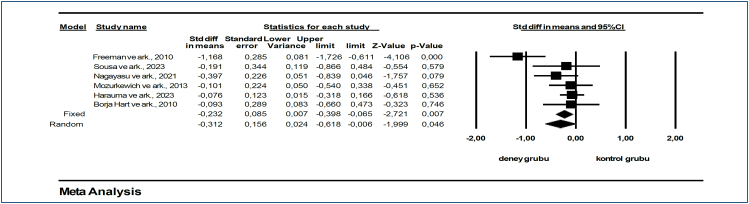
Forest plot for the effect of omega-3 fatty acids on depression in the intervention and control groups.

In this study, the presence of publication bias was determined using two methods: (a) funnel scatter plot and (b) Egger's regression test^20^. In the funnel plot, which is one of the important methods to show publication bias, we see that the studies in this data set are on the upper side of the funnel and show a symmetrical distribution. This shows us that there is no publication bias. Publication bias among the studies in this dataset was determined by Egger's method. According to Egger's method, the cut-off point (B0) is −2.07191, 95% confidence interval (-7.15761 to 3.01378), t=1.13112, df=4, and the two-way p-value is 0.32122. Figure 3 shows the effect sizes, standard error, variance, lower and upper limits, and forest plot of six studies on the effect of omega-3 fatty acid use on women's mental health in postpartum depression. Beck Depression Inventory (BDI), Edinburgh Postnatal Depression Scale (EPDS), and Hamilton Depression Rating Scale (HAM-D) were used to evaluate the effectiveness of mental health.

In a meta-analysis based on the findings of these studies, it was found that the use of omega-3 fatty acids in postpartum depression was effective on the depression level of women (SMD: −0, 312, 95%CI −0.618 to −0.006; Z=-1.999, p=0.046) (Figure 3). The collected data showed that omega-3 fatty acids had an overall significant effect on women's mental health, especially on the level of depression, in favor of the intervention group, and a moderate heterogeneity was found between the studies (I^2^=63.061%).

## DISCUSSION

This study was conducted to systematically review randomized controlled trials evaluating the effectiveness of omega-3 fatty acid use on women's mental health in postpartum depression and to conduct a meta-analysis of the available evidence.

According to the results of the analysis, it was determined that the use of omega-3 fatty acids decreased the level of PPD and positively affected women's mental health. Similar to our study, Zhang et al. reported that omega-3 fatty acids had ameliorative effects on PPD in their meta-analysis study^21^. In a randomized controlled study in Japan, Nagayasu et al. reported that the level of depression decreased in the experimental group using omega-3 fatty acids for 16 weeks^11^. We can say that omega-3 fatty acid consumption is effective in preventing or alleviating the effects of PPD. Firth et al. reported that omega-3 fatty acids have ameliorating effects on PPD when used in addition to pharmacological treatments^22^. It can be said that the results of this study do not have side effects of omega-3 acid use in addition to pharmacological treatments in the treatment of PPD. In studies in the literature, it has been determined that there is a relationship between low omega-3 fatty acid levels and PPD formation^12,17^. Low omega-3 fatty acid levels are an important risk factor that may cause PPD. It is important to have omega-3 fatty acid levels within normal limits for healthy mental health.

In a meta-analysis, Mocking et al. reported that omega-3 PUFAs have an overall significant benefit on perinatal depression^23^. In contrast to this study, Suradom et al. reported that omega-3 PUFAs did not have an overall significant benefit on perinatal depression in their meta-analysis^24^. The difference in the results is thought to be related to the difference in the intervention procedure and inclusion criteria. Therefore, it is thought that experimental studies should be conducted to obtain clearer results.

## CONCLUSION AND RECOMMENDATIONS

As a result of the study, it was found that the use of omega-3 fatty acids in postpartum depression was effective on women's mental health, especially depression levels. It is recommended that randomized controlled trials be conducted in groups with large sample sizes to investigate the effects of omega-3 fatty acids on women's mental health.

## Data Availability

The data that support the findings of this study are available from the corresponding author upon reasonable request.
